# Distribution of brain metastases: low-risk metastasis areas may be avoided when treating with whole-brain radiotherapy

**DOI:** 10.1186/s40644-020-00309-y

**Published:** 2020-04-22

**Authors:** Binwei Lin, Dan Huang, Xiyue Yang, Yu Zhang, Feng Gang, Xiao Bo Du

**Affiliations:** 1 Department of Oncology, Mianyang Central Hospital, Mianyang, Sichuan People’s Republic of China; 2 Radiology Department, Mianyang Central Hospital, Mianyang, Sichuan People’s Republic of China; 3 Department of Oncology, Affiliated Hospital of North Sichuan Medical College, Nan Chong, People’s Republic of China

**Keywords:** Brain metastasis, Whole-brain radiotherapy, Distribution

## Abstract

**Objective:**

Previous work has demonstrated that metastases are not uniformly distributed across the brain. This study aims to determine there are low-risk brain metastasis (BM) areas that may be avoided during whole-brain radiotherapy (WBRT) to reduce neurocognitive toxicity.

**Methods:**

Clinical and magnetic resonance imaging (MRI) data of 991 metastases in 192 patients with advanced cancer were analyzed retrospectively. Eleven anatomically defined regions of interest (ROIs) were contoured, and the locations of the BMs were recorded. Using the same definition, ROIs were contoured in 20 healthy volunteers.The proportions of patients with BMs in different ROIs, proportion of BMs, and proportion of different ROI volumes relative to the total volume were calculated.

**Results:**

The proportion of observed BMs was lower than expected in the brainstem, insula, diencephalon and internal structures, corpus callosum, and pituitary gland. The proportion of BMs was significantly higher than expected in the parietal lobe, occipital lobe, and cerebellum. For those patients with single BM, there was very low rate of low-risk ROIs involvement (0%), with 2–4 BMs, 6–13% of the patients had low-risk ROIs involvement, with ≥5 BMs, significant (> 30%) of the patients had low-risk ROIs involvement.

**Conclusion:**

The brainstem, insula, diencephalon and internal structures, corpus callosum, and pituitary gland demonstrate low risk for metastatic involvement. Involvement of low risk areas occurs in patients with more than 1 BM.

## Introduction

The incidence of brain metastasis (BM) is approximately 20–40% [1], and the most common primary cancer site is the lungs, accounting for approximately 50% of the primary cancer cases [2]. The prognosis of BMs is very poor. The median survival time is only 1–2 months when only steroids are used [3]. Presently, the National Comprehensive Cancer Network (NCCN) guidelines recommend surgery, radiation surgery, whole-brain radiotherapy (WBRT), and systemic therapy for BMs. Surgery and radiosurgery are mainly suitable for patients with limited BMs [4–6]. For patients with several lesions, WBRT is essential for treatment [7–9].

The application of new therapeutic methods, such as targeted therapy and immunotherapy, in the clinic setting has increased the survival time of patients with BM, bringing attention to issues regarding neurocognitive toxicity related to WBRT [10]. Radiation damage to the hippocampus is a key factor leading to cognitive decline [11]. The NRG-CC001 trial [12] reported that WBRT with hippocampal sparing could decrease neurocognitive toxicity compared with traditional WBRT. As the incidence of BMs within 5 mm around the hippocampus is only 4.7 to 8.6% [13–15], the limitation of the radiotherapy dose in the hippocampal area can decrease the neurocognitive toxicity related to WBRT without decreasing the intracranial tumor control rate [16].

Intracranial progression after radiotherapy is another common challenge. Bender et al. [17] reported that patients with BM from lung cancer and breast cancer had a higher risk of cerebellar metastasis. They found that a higher radiation dose (31.4–39.8 Gy) for the cerebellum and a lower dose (23.1–24.2 Gy) for other areas could achieve better local control.

Recently, Yanagihara et al. [18] conducted a study on 157 patients with BM. Fifty-five patients with anatomically defined ROIs were analyzed. They found that the brainstem, bilateral thalamus, hippocampus, parahippocampal gyrus, amygdala, and temporal pole showed BM involvement at a rate of 4.83%, representing additional low-risk areas. However, in Yanagihara’s study, the brain was divided into 55 ROIs, which may be difficult to apply in clinical practice. Moreover, the study only reported the incidence of BMs in different ROIs but did not report the proportion of patients with BMs in different ROIs. In this study, we divided the brain into 11 ROIs based on anatomy [19] and analyzed BMs in different ROIs to determine whether there are low-risk and high-risk ROIs associated with BMs.

## Materials and methods

### Patients

A single-center retrospective study was conducted. The study was approved by the institutional ethics committee (No: P2019022). Consecutive patients with first time diagnosis of BM between January 2015 and February 2019 were included. Sex, age, location of the primary tumor, pathological type, and number of BMs were recorded. The inclusion criteria were (1) primary pathology proven extracranial lesions; (2) age ≥ 18 years; (3) BMs diagnosed on MRI. The exclusion criteria were (1) no post-contrast images on MRI; (2) history of traumatic injury, neurosurgery, brain radiotherapy, cerebral hemorrhage, infarction, or infective diseases of the brain; and (3) history of primary brain neoplasms.

### Location of metastasis

**Fig. 1:**
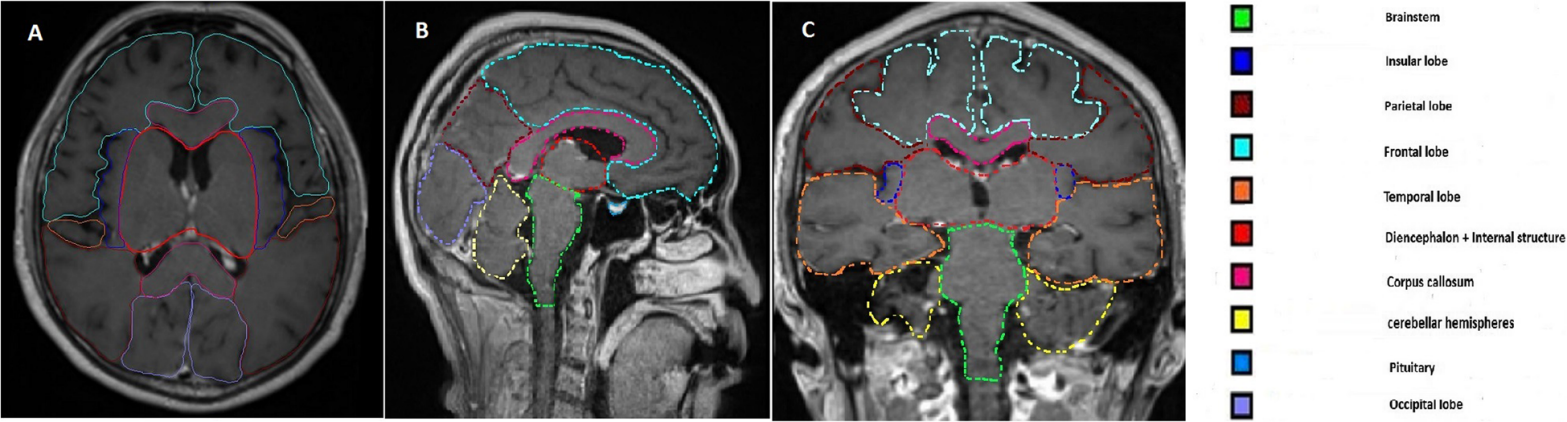
Delineation of Brain substructure on transaxial T1 weighted imaging (**a**),sagittal T1 weighted imaging (**b**) and coronal T1 weighted imaging (**c**)

Contrast-enhanced T1-weighted MRI (Siemens 3.0 T MAGNETOM Skyra MR/ Signa HDxt 1.5 T GEHCGEHC) scans of all patients were introduced into the Oncentra MasterPlan® treatment planning system (OTP, Version 4.2, Nucletron). A double dose of gadopentetate dimeglumine (Magnevist, 0.2 mmol/kg body weight) was administered intravenously 10 min before acquiring a 3-dimensional, coronal, inversion-recovery, spoiled-gradient, recalled acquisition sequence with 1.5 mm × 1.2 mm × 1.1 mm voxels (TR/ TE = 34 msec/4.7 msec; FOV 30 × 22 mm; matrix = 256 × 192; flip angle = 70°; 1.5 mm slices, scan time = 7–10 min). Eleven anatomically-defined ROIs, including the frontal lobe, parietal lobe, occipital lobe, temporal lobe, insula, internal structures (caudate nucleus, lentiform nucleus, and inner capsule), diencephalon, brainstem, cerebellar hemispheres, vermis, corpus callosum, and pituitary gland were contoured on the OTP [19] (Fig. 1).

The BMs were contoured on the OTP, and the locations of the BMs were recorded. If a BM involved two or more ROIs, the ROI where the largest BM volume was located was considered a positive anatomical area. The contours of the ROIs were independently drawn by two experienced radiation oncologists. When the ROI contours differed, a third radiation oncologist made the final decision.

We assumed that the proportion of BMs per unit volume is the same, and the proportion of expected BMs in ROI_A_ is:

$$ {\mathrm{V}}_{\mathrm{A}}/{\mathrm{V}}_{\mathrm{total}}\ast 100\% $$

where V_A_ is volume of ROI_A_ and V_total_ is total volume of all 11 ROIs).

As BMs have a space occupying effect which affects the brain ROI volume, we selected 20 patients with normal MRI results, contoured 11 ROIs according to the above method, and recorded the volume of ROIs to calculate the proportion of expected BMs. The ROIs of 20 normal brain MRI scans were also independently determined by two experienced radiation oncologists. The volume of different ROIs in the same patient was calculated as the average value of the two results.

### Statistical analysis

SPSS 22.0 software (IBM, Chicago, IL, USA) was used for statistical analysis. The observed and expected rate of BMs for each ROI were compared using the proportional two-tailed hypothesis test [20]. There was no multiple comparison correction in the statistical analysis process. A two-sided *P*-value of 0.05 or less was considered to indicate statistical significance.

## Results

### Clinical characteristics of patients

A total of 243 patients with BMs were included, of which 37 patients had only a head enhanced computed tomography examination and two patients had only a plain scan head MRI due to a contrast agent allergy and could not display the number and location of BMs accurately. In total, 204 patients with enhanced head MRI were imported into OTP, and 12 patients had indistinct MRI results due to motion artifact. Finally, 991 metastases in 192 patients with advanced cancer were analyzed in the study. The clinical characteristics of the patients are shown in Table 1. The mean age of all patients was 58 years. The proportion of men was 61.6%, and the most common primary tumor of BM was the lungs (75%). The average number of BMs for each patient was 5.1 and the median was 2.

**Table 1:** Basic clinical characteristics of included patients

Characteristic	***N*** = 192	***N*** = 20
**Age** (mean; years)	58.0	
**Sex** (mal; %)	118(61.6%)	
**Primary diagnosis**		
NSCLC	92	
SCLC	52	
Breast	14	
Unknown	7	
EC	5	
RC	5	
HCC	4	
CC	3	
OC	3	
Other	7	
**Number of metastasis**	991	
Mean	5.2	
Median	2	
IQR (25–75%)	1–5	
**Normal brain MRI**	–	20
Age (mean; years)	–	50.5
Sex (mal; %)	–	11(55%)

*NSCLC* non-small-cell lung cancer, *SCLC* small-cell lung cancer, *EC* esophageal cancer, *RC* rectal cancer, *HCC* hepatocellular carcinoma, *CC* colon cancer, *OC* ovarian cancer, *IQR* iInterquartile range

### Incidence of BM in different sites

The parietal lobe, occipital lobe, frontal lobe, cerebellum, and temporal lobe were common BM sites, and there were 115 (59.9%), 81 (42.2%), 80 (41.7%), 71 (37.0%), 51 (26.6%) patients with corresponding ROI metastasis, respectively. The proportion of observed BMs in the parietal lobe (31.7% vs. 14.5%, *P* < 0.001), occipital lobe (21.7% vs. 15.0%, *P* < 0.001), and cerebellum (16.6% vs. 10.7%, *P* < 0.001) was significantly higher than the proportion of expected BMs. The incidence of BMs in the brainstem, insula, diencephalon and internal structures, and corpus callosum was low, and there were 9 (4.7%), 7 (3.6%), 8 (4.2%) and 4 (2.1%) patients with metastasis, respectively. The proportion of observed BMs in the brainstem (1.0% vs. 2%, *P* < 0.02), insula (0.8% vs. 1.6%, *P* < 0.05), diencephalon and internal structures (0.8% vs. 5.3, *P* < 0.001), and corpus callosum (0.4% vs. 4.8%, *P* < 0.001) was significantly lower than the proportion of expected BMs, and the incidence of BMs located in pituitary was zero. The meninges were not an area of focus in this study; however, meningeal metastasis occurred in two patients.

**Fig. 2:**
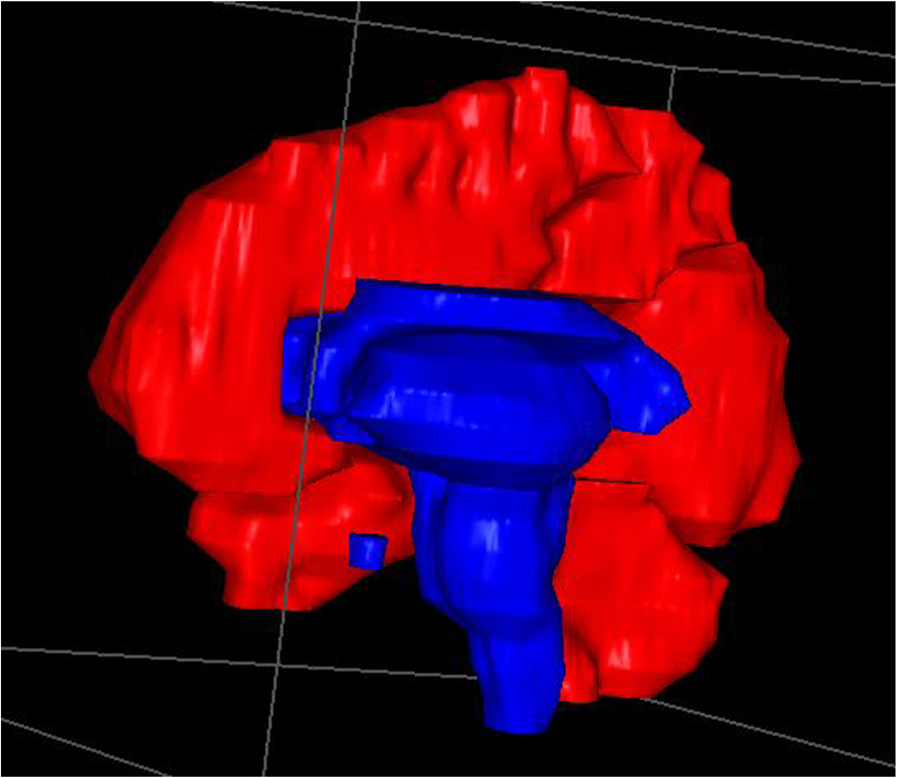
Low risk region of harboring a BM (blue area)

The low-risk ROIs (brainstem, insula, diencephalon and internal structures, corpus callosum, and pituitary) were analyzed as a single area (Fig. 2). The proportion of patients with BMs in this area was 13.0%, and the proportion of expected BMs in this area was 3.0%. The proportion of observed BMs in the low-risk ROIs was significantly lower than the proportion of expected BMs (3% vs. 13.8%, *P* < 0.001), Table 2. For those patients with single BM, there was very low rate of low -risk ROIs involvement (0%), with 2–4 BMs, 6–13% of the patients had low-risk ROIs involvement, with 5 or more BMs, significant (> 30%) of the patients had low-risk ROIs involvement (Table 3).

**Table 2:** Results from the Analysis of different brain ROIs

Location of metastasis	***N*** = 192 patients	***N*** = 991 metastases	Structure Volume mean ± SD (V_**A**_/ V _total_)cm^**3**^	***P*** value^b^
Temporal lobe	51 (26.6%)	95 (9.6%)	192.3 ± 9.7 (16.1%)	< 0.001
Left	34 (17.7%)	47 (4.7%)	96.4 ± 4.9 (8.1%)	
Right	35 (18.2%)	48 (4.8%)	96.0 ± 5.0 (8.0%)	
Frontal lobe	80 (41.7%)	170 (17.2%)	358.3 ± 12.8 (30.0%)	< 0.001
Left	53 (27.6%)	82 (8.3%)	179.3 ± 6.0 (15.0%)	
Right	55 (28.6%)	88 (8.9%)	179.1 ± 7.1 (15.0%)	
Parietal lobe	115 (59.9%)	314 (31.7%)	173.1 ± 15.5 (14.5%)	< 0.001
Left	83 (43.2%)	168 (17.0%)	86.8 ± 7.3 (7.3%)	
Right	74 (38.5%)	146 (14.7%)	86.3 ± 8.3 (7.2%)	
Occipital lobe	81 (42.2%)	215 (21.7%)	179.1 ± 9.0 (15.0%)	< 0.001
Left	53 (27.6%)	100 (10.1%)	89.3 ± 4.7 (7.5%)	
Right	57 (29.7%)	115 (11.6%)	89.7 ± 4.4 (7.5%)	
Cerebellum	71 (37.0%)	165 (16.6%)	128.4 ± 8.9 (10.7%)	< 0.001
Left	47 (24.5%)	77 (7.8%)	59.3 ± 4.3 (5.0%)	
Right	42 (21.9%)	74 (7.5%)	59.3 ± 5.0 (5.0%)	
Vermis	12 (6.3%)	14 (1.4%)	9.8 ± 0.8 (0.8%)	
Brainstem	9 (4.7%)	10 (1.0%)	24.5 ± 1.9 (2.0%)	< 0.02
Left	5 (2.6%)	5 (0.5%)	–	
Right	5 (2.6%)	5 (0.5%)	–	
Insular lobe^c^	7 (3.6%)	8 (0.8%)	19.1 ± 2.0 (1.6%)	< 0.05
Left	6 (3.1%)	6 (0.6%)	9.6 ± 1.1 (0.8%)	
Right	2 (1.0%)	2 (0.2%)	9.5 ± 1.0 (0.8%)	
Diencephalon + Internal structure^a^	8 (4.2%)	8 (0.8%)	63.1 ± 8.1 (5.3%)	< 0.001
Corpus callosum	4 (2.1%)	4 (0.4%)	57.8 ± 7.0 (4.8%)	< 0.001
Pituitary	0	0	0.7 ± 0.1 (0.1%)	> 0.2
Brainstem+ Insular lobe+ Diencephalon Internal structure^a^ + Corpus callosum + pituitary	25 (13.0)	30 (3%)	165.1 ± 9.7 (13.8%)	< 0.001

*ROIs* regions of interest, *VA* volume of ROIA, *Vtotal* total volume of all 11 ROIs

^a^Internal structure Include caudate nucleus, lentiform nucleus, inner capsule

^b^Brain metastasis ratio vs Volume ratio

^c^Insular lobe Include Insular cortex, external capsule, extreme capsule, claustrum

**Table 3:** The proportion of patients with involvement of the low-risk ROIs given different cutoffs of BMs

Cutoffs of BMs	Number of Patients with BMs in low-risk ROIs given specific cutoff of BMs (***N*** = 25)	Number of Patients with specific cutoff of BMs (***N*** = 192)	Proportion
1	0	74	0 (0/74)
2	4	32	12.5% (4/32)
3	1	15	6.7% (1/15)
4	2	16	12.5% (2/16)
≥5	18	55	32.7% (18/55)

*BMs* brain metastases, *ROIs* regions of interest

## Discussion

The main purpose of this study was to determine the low-risk and high-risk BM areas through the analysis of metastasis sites of newly-diagnosed patients with BM to provide the basis to prevent low-risk areas and increase the irradiation dose of the high-risk areas during WBRT. Some studies [18, 20] have reported a difference in the risk of metastasis in different sub-structures of the brain; however, these studies divided the brain into several ROIs, thus, making it difficult to apply this approach in clinical practice. In this study, the whole brain was divided into 11 structures: the frontal lobe, parietal lobe, occipital lobe, temporal lobe, insula, internal structures and the diencephalon, brainstem, cerebellar hemispheres, vermis, corpus callosum, and pituitary. We found that the incidence of BM in the brainstem, insula, diencephalon and internal structures, and corpus callosum was low, and there was no BM in the pituitary; however, the incidence in the parietal lobe, cerebellum, and occipital lobe was high.

Through the integrated analysis of the brainstem, insula, diencephalon and internal structures, corpus callosum, and pituitary, we found that both the proportion of patients with BMs in this region and the expected proportion of BMs in this region increased, which may be caused by the increase of volume when all low-risk areas are analyzed as a whole. However, we found that the proportion of observed BMs in the brainstem, insula, diencephalon and internal structures, and corpus callosum was significantly lower than the proportion of expected BMs, and the proportion of observed BMs in the parietal lobe, cerebellum, and occipital lobe was significantly higher than the proportion of expected BMs. Therefore, our previous assumption that “the opportunity of BMS per unit volume is the same” is wrong. Volume may be only one of the reasons that affect the distribution of brain metastasis, and there may be other reasons, such as differences in pathological types and primary tumors [21–23]. In Yanagihara’s [20] research, the brains were divided into 52 ROIs. There were significant differences between the proportion of observed BMs and the proportion of expected BMs in 10 regions, including the frontal pole, lateral occipital cortex, and middle frontal gyrus. The main reason for this difference from our results may be that the occupying effect of the metastases would affect the volume ratio. Therefore, we obtained this based on a normal brain, even though Yanagihara used patients with BMs.

We found that the proportion of observed BMs in low-risk ROIs was only 3.0%, significantly lower than the proportion of expected BMs. Yanagihara et al. [18] also found that the brainstem, bilateral thalamus, hippocampus, parahippocampal gyrus, amygdala, and temporal pole showed BM involvement at a rate of 4.83%, and these low-risk ROIs may be avoided when treating with WBRT. However, Yanagihara et al. did not report the proportion of patients with BMs in their low-risk ROIs. In our study, we found that the proportion of patients with BMs in low-risk ROIs reached 13.0%. If the low-risk ROIs are avoided during WBRT, more than 10% of patients may relapse. Table 3 showed that when the number of BMs ≥5, more than 30% of the patients had low risk ROIs involvement. Therefore, WBRT may not avoid whole low-risk ROIs in patients with BMs ≥5. These low-risk ROIs important to the protection of neurocognitive and endocrine functions need to be further studied so as to avoid these ROIs in patients with BMs ≥5.

When the number of BMs < 5, the possibility of BMs in low-risk areas may be low, especially for those patients with single BM, We didn’t find metastasis in the low-risk areas, which makes it possible to protect these areas when performing WBRT. However, a “low-risk” area of the brain might only be low risk for metastasis failure, but not necessarily low risk clinically. Even though the incidence of brain metastasis of these important structures may be very low, once there is a metastasis, it is often fatal. Therefore, whether these areas can be avoided during WBRT requires more rigorous prospective randomized controlled clinical trials. We found that the incidence of BMs in the parietal lobe, cerebellum, and occipital lobe was higher than the corresponding ROI volumes relative to the total volume of all 11 ROIs. The parietal lobe, cerebellum, and occipital lobe were high-risk for having BMs, which was also confirmed in other studies. Bender et al. [17] found that the proportion of BMs in the cerebellum is high. Quattrocchi et al. [24] found that non-small cell lung cancer has a high incidence of BM in the parietal lobe, occipital lobe, and cerebellum, while breast cancer had a high incidence of BM in the cerebellum.

The limitations of this study are as follows: (1) regarding patient selection, we noticed that NRG-CC001 did not include small cell lung cancer (SCLC) patients because of the possibility of diffuse intracranial metastasis of SCLC. Our study is a retrospective study aiming to describe the probability of brain metastases in different regions of the brain regardless of primary tumor histology, so we included patients with SCLC. We divided the pathological types into three categories: small cell lung cancer, non-small cell lung cancer, and others; 13, 15.3, and 10.4%, respectively, of the patients had low-risk areas metastasis, and there was no significant difference between the three groups (*P* = 0.762). (2)The hippocampus area has been confirmed to be an area with a low-risk of developing BM [13], and research has confirmed that protecting this area can reduce the cognitive decline caused by WBRT [12]. Therefore, we did not conduct a separate BM rate analysis of the hippocampus, which is one of the shortcomings of this study. (3) Furthermore, this study did not report the relationship between the dose of radiotherapy and side effects in this area. Because this is a retrospective study, the scanning thickness of brain MRI in our hospital was 4–5 mm. Still, some benign lesions are difficult to distinguish from BMs, and MRI may misdiagnose or miss BMs. (4) We defined that if a BM involved two or more ROIs, the ROI where the largest BM volume was located was considered to be a positive anatomical area. This may be justified when doing volumetric analysis, but this would be problematic when looking at the involved ROI as the radiation field has to cover the entire lesion and marginal zone. This was not considered in our study. However, there was no brain metastasis involving both low-risk and high-risk areas during data collection.

## Conclusions

We found that the brainstem, insula, diencephalon and internal structures, corpus callosum, and pituitary are low risk areas for BMs, which may be avoided when treating with WBRT, but WBRT may not necessarily avoid low-risk ROIs in patients with BMs ≥5. Future studies are needed to confirm whether the avoidance of those ROIs on WBRT can reduce the incidence of neurocognitive and endocrine toxicity under the condition of similar intracranial tumor control rates.

## Data Availability

Not applicable.

## Abbreviations

BM: Brain metastasis
WBRT: Whole-brain radiotherapy
MRI: Magnetic resonance imaging
ROIs: Regions of interest
NCCN: National Comprehensive Cancer Network
RTOG: Radiation Therapy Oncology Group
OTP: Oncentra MasterPlan® treatment planning system
